# Integrated Dissection of lncRNA-miRNA-mRNA Pairs and Potential Regulatory Role of lncRNA PCAT19 in Lung Adenocarcinoma

**DOI:** 10.3389/fgene.2021.765275

**Published:** 2022-01-12

**Authors:** Xiaomei Tang, Xiaoyan Hua, Xujin Peng, Yongyan Pei, Zhigang Chen

**Affiliations:** ^1^ Jiangxi Chest Hospital, Nanchang, China; ^2^ Department of Oncology, Wannian County Hospital of Traditional Chinese Medicine, Shangrao, China; ^3^ School of Medicine and Chemical Engineering, Guangdong Pharmaceutical University, Guangzhou, China; ^4^ Department of Oncology, Shangrao People’s Hospital, Shangrao, China

**Keywords:** lung adenocarcinoma, lncRNA, proliferation, migration, invasion

## Abstract

Lung adenocarcinoma (LUAD) is the main cause of cancer-related deaths worldwide. Long noncoding RNAs have been reported to play an important role in various cancers due to their special functions. Therefore, identifying the lncRNAs involved in LUAD tumorigenesis and development can help improve therapeutic strategies. The TCGA-LUAD RNA expression profile was downloaded from The Cancer Genome Atlas, and a total of 49 differential lncRNAs, 112 differential miRNAs, and 2,953 differential mRNAs were screened. Through Kaplan–Meier curves, interaction networks, hub RNAs (lncRNAs, miRNAs, and mRNAs) were obtained. These hub genes are mainly involved in cell proliferation, cell cycle, lung development, and tumor-related signaling pathways. Two lncRNAs (SMIM25 and PCAT19) more significantly related to the prognosis of LUAD were screened by univariate Cox analysis, multivariate Cox analysis, and risk model analysis. The qPCR results showed that the expression levels of SMIM25 and PCAT19 were downregulated in clinical tissues, A549 and SPC-A1 cells, which were consistent with the bioinformatics analysis results. Subsequently, the PCAT19/miR-143-3p pairs were screened through the weighted gene co-expression network analysis and miRNA-lncRNA regulatory network. Dual luciferase detection confirmed that miR-143-3p directly targets PCAT19, and qPCR results indicated that the expression of the two is positively correlated. Cell function tests showed that overexpression of PCAT19 could significantly inhibit the proliferation, migration, and invasion of A549 and SPC-A1 cells. In contrast, knockout of PCAT19 can better promote the proliferation and migration of A549 and SPC-A1 cells. The expression of PCAT19 was negatively correlated with tumor grade, histological grade, and tumor mutation load in LUAD. In addition, co-transfection experiments confirmed that the miR-143-3p mimic could partially reverse the effect of PCAT19 knockout on the proliferation of A549 and SPC-A1 cells. In summary, PCAT19 is an independent prognostic factor in patients with LUAD that can regulate the proliferation, migration, and invasion of LUAD cells and may be a potential biomarker for the diagnosis of LUAD. PCAT19/miR-143-3p plays a very important regulatory role in the occurrence and development of LUAD.

## Introduction

Lung cancer is one of the most common malignant tumors in the world, of which non–small cell lung cancer (NSCLC) is the main subtype (Gandhi et al., 2018; Ferlay et al., 2019; Cai and Liu, 2021). Furthermore, lung adenocarcinoma (LUAD) is the most common NSCLC and accounts for a large proportion of cancer-related deaths worldwide (Liu et al., 2019; Siegel et al., 2019). Despite great efforts to improve the treatments and prognosis of patients with LUAD, the average 5-year survival rate is still under 20% (Kim et al., 2015; Lin et al., 2016). The leading causes of this phenomenon are cancer cell metastasis and chemoresistance (Alvarado-Luna and Morales-Espinosa, 2016; Chen et al., 2019). Therefore, an intensive study on the molecular mechanism of LUAD progression is required to identify new biomarkers and targeted therapies.

**GRAPHICAL ABSTRACT:**
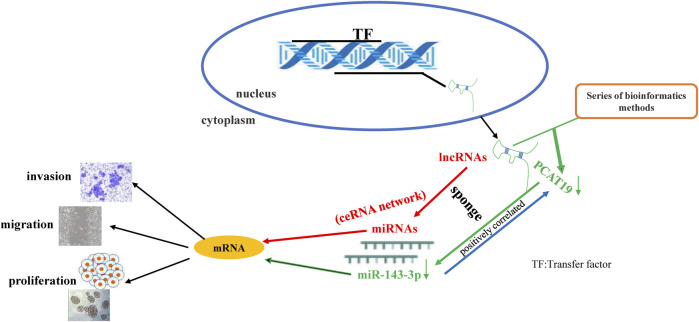
Our data revealed that PCAT19 regulates the proliferation, migration, and invasion of lung cancer cells by targeting the miR-301b-3p. The red arrow, red line, and red font indicate that the progression of lung cancer cells can be regulated through an lncRNA/miRNA/mRNA regulatory network. The green arrow, green line, and green font represent the PCAT19/miR-143-3p/mRNA regulatory network we screened and analyzed in this project. Black arrows, black lines, and black fonts represent the common regulation process of the two situations in the figure. TF, transfer factor.

Long noncoding RNAs (lncRNAs) are noncoding RNAs that are more than 200 nucleotides in length (Peng et al., 2017). Emerging studies have confirmed that lncRNAs are abnormally expressed in the biological processes of various cancers, including breast cancer (Liang et al., 2020), lung cancer (Lai et al., 2020), esophageal cancer (Zhang et al., 2020), and gastric cancer (He et al., 2019). MicroRNAs (miRNAs) are small endogenous noncoding RNAs that play an important role in regulating gene expression, and their regulatory networks are involved in many biological processes (Ali Syeda et al., 2020). Salmena et al. (2011) proposed a competing endogenous RNA (ceRNA) hypothesis, the core concept of which is that ceRNAs interact with target miRNAs through miRNA response elements to control the transcriptome on a large scale. The cross-talk between ceRNAs is achieved by lncRNA-miRNA-mRNA networks. This delicate and complex regulatory network may contribute to a more precise understanding of the disease process (Wang et al., 2019; Zhou et al., 2019; Ma et al., 2020). However, lncRNA-miRNA-mRNA networks have not been sufficiently studied in human cancers, including LUAD. Over the past few years, an increasing number of studies have shown the regulation of lncRNAs in LUAD (Dong et al., 2018; Guo et al., 2019; Jia et al., 2019; Ying et al., 2020). These lncRNAs participate in multiple biological processes, such as cell proliferation, cell cycle, cell migration, and drug resistance by different regulatory mechanisms (Cheng et al., 2019; Zhu et al., 2019). Therefore, studies on lncRNAs may be of enormous value in understanding the occurrence and development of tumors. Although many lncRNAs have been reported in numerous tumors, still many lncRNAs are to be investigated.

In this study, we aimed to identify new therapeutic targets and prognostic biomarkers for LUAD. The RNA expression profile of LUAD in The Cancer Genome Atlas (TCGA) was analyzed to identify the differentially expressed lncRNAs, miRNAs, and mRNAs, and Gene Ontology (GO), Kyoto Encyclopedia of Genes and Genomes (KEGG), Kaplan–Meier, Cox regression, gene set enrichment analysis (GSEA), ceRNA, protein–protein interaction (PPI), and other bioinformatics analyses were used to determine the functions of differentially expressed hub lncRNAs. At the same time, we detected the expression of hub lncRNAs in LUAD tissues and A549 cells and explored the effects of hub lncRNAs and their coaction on A549 cells through functional loss and gain experiments, dual luciferase experiments, and co-transfection experiments.

## Materials and Methods

### Screening of Differentially Expressed RNAs

The RNA expression profile of LUAD was derived from TCGA, containing sequencing data of 585 samples (Table 1). Clinical information (age, race, survival time, survival status, tumor grade, and histological grade) of all samples was also downloaded. The limma package of R was used to analyze the differential expression profile. The screening criteria were *p* < 0.01, false discovery rates (FDR) < 0.05, and |log_2_ fold change |>1.

**TABLE 1:** Clinical pathological characteristics of 585 patients with lung adenocarcinoma.

Parameter	Subtype	Patients (%)
Age (years)	33 to ≤ 66	287 (49.06%)
	66 to ≤ 89	298 (50.94%)
Gender	Male	316 (54.02%)
	Female	269 (45.98%)
Pathologic stage	stage i	316 (54.02%)
	stage ii	135 (23.08%)
	stage iii	97 (16.58%)
	stage iv	37 (6.32%)
Pathologic T	T1	191 (32.65%)
	T2	321 (54.87%)
	T3	50 (8.55%)
	T4	23 (3.93%)
Pathologic M	M0	424 (72.48%)
	M1	161 (27.52%)
Pathologic N	N0	376 (64.27%)
	N1	113 (19.32%)
	N2	91 (15.56%)
	N3	5 (0.85%)
Vital status	Alive	365 (62.39%)
	Dead	220 (37.61%)

### Kaplan–Meier Survival Analysis of lncRNAs

To explore whether the expression level of differentially expressed lncRNAs has an effect on the prognostic survival of patients with LUAD, GEPIA 2.0 (http://gepia2.cancer-pku.cn) was used to perform Kaplan–Meier survival analysis on all differentially expressed lncRNAs. *p* < 0.05 was judged as statistically significant. Then, Starbase 2.0 (http://starbase.sysu.edu.cn/) software was used to verify the data obtained from the analysis.

### Constructing the lncRNA-miRNA-mRNA ceRNA Network

To construct the ceRNA network, we used Starbase 2.0 software to verify the regulatory correlation between miRNA-mRNA and miRNA-lncRNA. Then, the differentially expressed RNAs (lncRNAs, miRNAs, and mRNAs) were compared to obtain overlapping RNAs (lncRNAs, miRNAs, and mRNAs). The network was constructed and observed using Cytoscape.

### Protein–Protein Interaction Network Construction of mRNAs in the ceRNA Network

To identify relevant hub genes, the search tool for the Retrieval of Interacting Genes/Proteins (STRING 11.5, https://string-db.org/) was utilized. The PPI was analyzed using the STRING database, and a combined score>0.4 was used as the cutoff criterion. Additionally, Cytoscape 3.9 was used to construct and visualize the PPI network.

### Gene Ontology and Kyoto Encyclopedia of Genes and Genomes Analysis of mRNAs in the ceRNA Network

The functional enrichment analysis of mRNAs in the ceRNA network was performed using Metascape (http://metascape.org/). All statistically enriched terms (GO and KEGG) were identified based on accumulative hypergeometric *p*-values.

### Cox Proportional Regression Model Based on the Expression of Hub lncRNAs

To analyze the independent influence of individual lncRNAs on the overall survival of patients with LUAD, we used an online tool (Sangerbox tools 3.0, http://sangerbox.com/Tool) to perform univariate and multivariate Cox proportional regression analysis. Based on the analysis results, we established a risk regression model to further verify the clinical value of lncRNAs. The risk model formula is as follows: a × exp (lncRNA1) + a × exp (lncRNA2) + ……+ a × exp (lncRNAn), where a represents the multivariate Cox regression coefficient and exp () represents the expression level of lncRNA. Then, based on the risk value, the patients were divided into high- and low-risk groups, the risk curves of the two groups were calculated, and their 1-, 3-, and 5-year ROC (receiver operating characteristic) curves were drawn to test the predictive ability of the model.

### Weighted Gene Co-Expression Network Analysis of miRNAs in GSE74190

The LUAD dataset GSE74190 in the GEO database was used to perform the weighted gene co-expression network analysis (WGCNA). We first remove sample outliers based on the thresholding power value and built a sample tree. The Pearson correlation coefficient was used to calculate the correlation between gene expression, and the correlation matrix of gene expression data was constructed. Then, the matrix was converted to a connection matrix to calculate the topological overlap matrix (TOM). Subsequently, gene modules were established, the minimum module size of each module was set to 30, and the correlation between the module and the trait was calculated based on the data of each module. Finally, TOM-based dissimilarity was used for cluster analysis and the Pearson correlation coefficient was used to analyze the relationship between LUAD in each module and normal tissues.

### Gene Set Enrichment Analysis

We used the standardized expression profile data obtained from the LUAD dataset in TCGA for GSEA. The number of permutations was set to 1,000. Using GSEA, we analyzed the GO and KEGG pathways to study the possible biological functions of PCAT19. *p* < 0.05 indicates that the analysis result is meaningful.

### Cell Lines

A549, SPC-A1, and 16HBE cells used in this study were acquired from the Institute of Beina Chuanglian Biotechnology Research, Beijing, China. A549 and 16HBE cells were cultured in RPMI-1640 medium (Invitrogen, United States), and 10% fetal bovine serum (FBS, Gibco, United States) was used to supplement the medium with 100 mg ml^−1^ streptomycin and 100 U ml^−1^ penicillin. SPC-A1 cells were cultured in Dulbecco-modified Eagle medium (Invitrogen, United States), and 10% fetal bovine serum (FBS, Gibco, United States) was used to supplement the medium with 100 mg ml^−1^ streptomycin and 100 U ml^−1^ penicillin. The medium was kept in incubators at 37°C with 5% CO_2_.

### RNA Extraction and qRT–PCR

A total of 30 LUAD and 30 adjacent tissues were collected (Supplementary Material S1). This study was approved by the hospital ethics committee and was carried out in accordance with the Declaration of Helsinki. In addition, each patient provided written informed consent. All the tissues were stored at 80°C immediately after surgical resection.

Total RNA was extracted using the RNeasy Mini Kit (Qiagen, Germany) according to the manufacturer’s instructions. The concentration of the extracted RNA was measured with Nanodrop. The same amount of RNA was reverse transcribed into cDNA using the Golden star^TM^ RT6 cDNA synthesis kit (TsingKe, Beijing). Then, the Master qPCR Mix (SYBR GREEN 1) (Qingdao, Beijing) was used to perform qRT-PCR to detect the expression of RNAs. The expression of GAPDH was used as a reference for lncRNA, and U6 was used as a reference for miRNA. The formula 2^−ΔΔCt^ was used to calculate the expression levels of lncRNAs and miRNAs. Primer 5.0 software (SMIM25: Forward (5′-3′)-TCT​CTG​GGT​GGA​ATG​TCA​C, Reverse (5′–3′)-TTT​ACT​GGG​CAC​TTG​TCC​T; PCAT19: Forward (5′-3′)-CCA​ATG​ACA​TCC​AAT​GGA​GG, Reverse (5′–3′)-TCC​TGG​TGG​TTG​TTT​AAT​CAC) was used for the lncRNA primer design, and miRNA primers (miR-143-3p: MQPS0000635-1-100) were purchased from Guangzhou RiboBio. In addition, the qRT-PCR data were analyzed by Excel. The two groups were tested by *t*-test (*p* < 0.05).

### Cell Transfection

Si-PCAT19 and the corresponding siRNA control (si-NC) were purchased from Guangzhou RiboBio. The PCAT19 overexpression plasmid (pCDH-GFP-PCAT19) and the corresponding control plasmid (NC) were also purchased from Guangzhou RiboBio. All plasmids were tested according to the manufacturer’s instructions and were transfected into lung cancer cells (A549 and SPC-A1 cells) using the Lipofectamine 3000 reagent (Invitrogen, United States).

### Cell Counting Kit-8 Assay

The proliferation of lung cancer cells (A549 and SPC-A1 cells) was evaluated by the cell counting kit-8 (CCK-8) assay (Beyotime, Beijing) according to the manufacturer’s instructions. Lung cancer cells were collected and then seeded in 96 wells at 1 × 10^4^ cells/well with culture medium at 37°C with 5% CO_2_. Cells were then subjected to culture for 24 and 48 h before the addition of 10 μl of CCK-8 (5 mg/ml) to each well. After 3 h of incubation, a multimode reader (Thermo, United States) was utilized to detect the absorbance at 450 nm of each well. Each experiment was carried out three times.

### Wound-Healing Assay

Lung cancer cells (A549 and SPC-A1 cells) were collected and then seeded in 96 wells at 5 × 10^5^ cells/well with culture medium at 37°C with 5% CO_2_. Cells were cultured to a confluent monolayer, which was scratched by a sterile pipette tip (200 μl). Then, the cells were flushed twice with PBS and subjected to reduced serum medium, and photos were taken after 0, 12, 24, and 48 h. The scratched areas were recorded by a microscope (Olympus, Japan), and ImageJ software was utilized to evaluate the percentage of closure.

### Transwell Assay

After 48 h of transfection, lung cancer cells (A549 and SPC-A1 cells) were collected to prepare a single cell suspension. The lung cancer cell suspension (3 × 10^3^ cells/well) was added to the upper chamber (Corning, United States), and the medium (20% fetal bovine serum) was added to the lower chamber. Matrigel was added to the upper chamber. After 24 h, the cells were fixed with 4% paraformaldehyde (Sigma, United States) and stained with 1% crystal violet (Sigma, United States). Cell observation and counting were performed under a light microscope (Olympus, Japan).

### Soft Agar Colony Formation Assay

A549 and SPC-A1 cells were seeded on a six-well culture plate at 3 × 10^5^ cells per well, and the plasmid was transfected at 70% cell confluence. After 24 h of transfection, the cells of each group were collected for viable cell count, and the cell density was adjusted to 2000 cells/ml with medium containing 20% FBS and then maintained in a medium containing 10% FBS, 100 μg/ml streptomycin, 100 units/ml penicillin, 0.7% soft agar, and 1.2% soft agar. The medium was incubated at 37°C with 5% CO_2_ in an incubator. After 14 days, the colonies were fixed with 4% paraformaldehyde at room temperature and then stained with 0.005% crystal violet. The number of clones larger than 0.05 mm and the number of total clones in the field of view were manually counted under the microscope: the clone formation rate = the number of clones larger than 0.05 mm/the number of all clones × 100%.

### Luciferase Reporter Assay

A549 cells were seeded into 96-well plates and were co-transfected with 60 ng of the dual luciferase reporter construct pmiR-RB-Report^™^-PCAT19 3′UTR (RiboBio, Guangdong) and miR-143-3p mimic. After 48 h of incubation, the fluorescence value was measured by the dual luciferase reporter kit (Promega, United States).

### Statistical Analysis

GraphPad Prism 8 and SPSS 25.0 (California, CA) were utilized to perform statistical analysis. The differences were deemed statistically significant at *p* < 0.05.

## Result

### Aberrantly Expressed lncRNAs in Lung Adenocarcinoma

**FIGURE 1:**
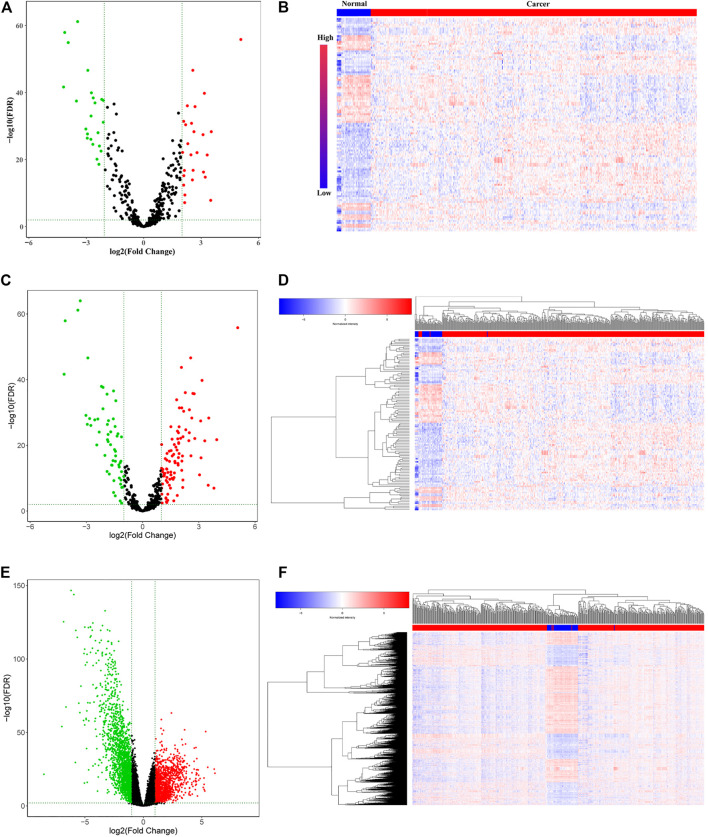
Differentially expressed RNAs (lncRNAs, miRNAs, and mRNAs) in the RNA expression profile of lung adenocarcinoma in TCGA. **(A)** Volcano plot and **(B)** heatmap for the differentially expressed lncRNAs. **(C)** Volcano plot and **(D)** heatmap for the differentially expressed miRNAs. **(E)** Volcano plot and **(F)** heatmap for the differentially expressed miRNAs. The red dots represent upregulated genes, and the green dots represent downregulated genes. The black dots represent genes with no significant difference.

To identify the hub lncRNAs involved in the progression of LUAD, we downloaded the LUAD expression profile data from TCGA and performed differential analysis. A total of 26 upregulated lncRNAs and 23 downregulated lncRNAs were identified (Figures 1A,B; Table 2). Then, the screened differentially expressed lncRNAs were analyzed comprehensively. The Kaplan–Meier curve analysis revealed 14 lncRNAs associated with the prognosis of LUAD patients (Supplementary Figure S1; Table 2), indicating that these lncRNAs may play an important role in the occurrence of LUAD. However, the function of most lncRNAs in LUAD requires further study.

**TABLE 2:** Prognosis values of differentially expressed long noncoding RNAs in lung adenocarcinoma.

lncRNA	log_2_ FC	P Adj	Overall survival (*p*-value)
FAM83A-AS1	5.33	5.31E-53	3.90E-05^a^
AFAP1-AS1	5.25	1.91E-11	5.60E-01
Z98257.1	4.73	1.63E-22	6.00E-01
FEZF1-AS1	4.54	3.36E-24	3.70E-01
MNX1-AS1	4.36	1.52E-41	1.30E-01
ZFPM2-AS1	4.35	6.81E-27	9.60E-02
AL391056.1	3.39	4.72E-27	4.20E-01
LINC00511	3.05	1.41E-26	6.30E-02
AC025580.1	3.03	3.97E-21	2.90E-01
AL354719.2	3.01	1.18E-20	3.10E-01
AL365181.3	2.96	1.76E-11	1.20E-02^a^
LINC01270	2.54	6.39E-31	7.00E-01
LUCAT1	2.52	1.92E-12	5.70E-01
AC004816.1	2.52	1.12E-34	3.30E-01
VPS9D1-AS1	2.50	6.26E-24	6.80E-03^a^
BLACAT1	2.48	6.84E-16	2.20E-01
U62317.2	2.47	1.18E-25	3.20E-01
PVT1	2.44	5.19E-28	6.10E-01
AL354707.1	2.38	8.11E-19	8.70E-01
PCAT6	2.35	2.83E-26	4.50E-01
LINC01426	2.26	4.86E-18	3.80E-01
LINC02362	2.16	3.51E-17	6.80E-02
AL157838.1	2.10	4.93E-28	7.50E-02
AL355312.3	2.04	6.22E-13	5.20E-01
LINC00857	2.04	3.84E-26	6.10E-02
AL445524.1	2.01	2.63E-16	1.20E-01
AC011511.5	−2.02	4.65E-24	3.70E-01
AC144831.1	−2.06	1.21E-42	5.80E-01
TBX5-AS1	−2.06	5.63E-47	1.70E-02^a^
AF131215.5	−2.18	2.96E-38	8.10E-03
MBNL1-AS1	−2.24	2.70E-69	5.60E-02
AC125807.2	−2.27	4.84E-72	3.20E-01
AC010329.1	−2.35	6.31E-16	8.00E-04^a^
AC116407.1	−2.53	6.69E-52	5.30E-02
AL162511.1	−2.55	8.17E-22	2.50E-03^a^
LINC00261	−2.60	2.46E-19	3.70E-02^a^
AC011899.2	−2.62	5.48E-81	1.60E-01
PCAT19	−2.70	1.52E-101	1.60E-04^a^
SMIM25	−2.76	6.05E-79	3.30E-02^a^
RHOXF1-AS1	−2.77	4.41E-34	3.80E-02^a^
HHIP-AS1	−2.78	1.36E-33	8.40E-02
LINC01936	−2.83073824	1.72E-63	1.40E-01
C8orf34-AS1	−2.83225284	6.53E-25	2.80E-02^a^
SFTA1P	−3.231128408	2.64E-42	1.90E-01
AC079630.1	−3.496028269	5.49E-51	1.70E-02^a^
AC093110.1	−3.601261229	2.22E-116	2.7E-01
LHFPL3-AS2	−4.083740091	1.30E-54	4.80E-03^a^
FENDRR	−4.550706947	1.41E-118	2.60E-03^a^
AC008268.1	−5.015890223	1.37E-57	2.00E-01

a Represents prognostic lncRNAs.

### Construction of the lncRNA-miRNA-mRNA Network in Lung Adenocarcinoma

**FIGURE 2:**
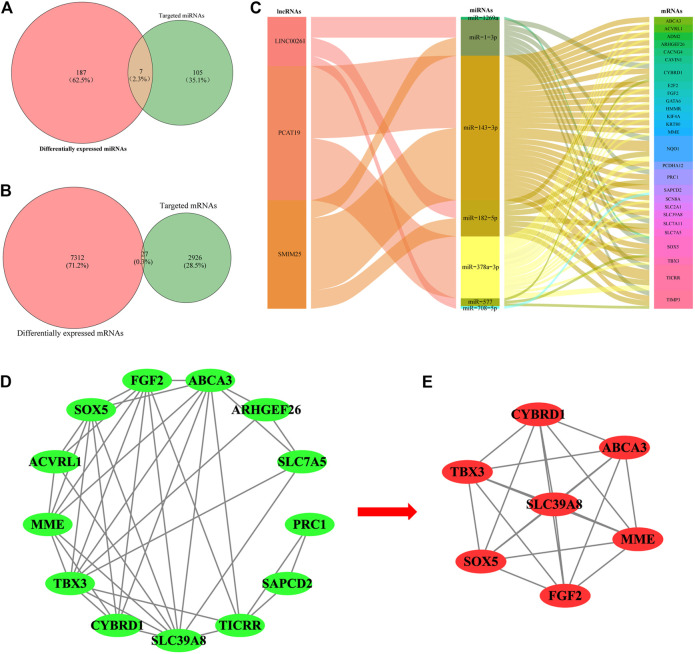
Venn diagram of the common **(A)** miRNAs and **(B)** mRNAs between differentially expressed miRNAs/mRNAs and target miRNAs/mRNAs. **(C)** Sankey diagram of the ceRNA network in lung adenocarcinoma. Each rectangle represents a gene, and the degree of connectivity of each gene is indicated by the size of the rectangle. **(D)** PPI network of the lncRNA-correlated mRNAs in the ceRNA network. **(E)** Most significant module in the PPI network of the lncRNA-correlated mRNAs by the MCODE plug-in.

The limma package was used to analyze the expression profile data of LUAD from TCGA and to analyze the differentially expressed miRNAs and mRNAs according to the adjusted *p* < 0.01, FDR<0.05, and |log_2_ fold change |>1 screening criteria. A total of 112 differentially expressed miRNAs (70 upregulated and 42 downregulated) (Figures 1C,D; Supplementary Table S1) and 2,953 differentially expressed mRNAs (1,097 upregulated and 1,856 downregulated) (Figures 1E,F; Supplementary Table S2) were identified. Then, the Starbase tool was used to analyze the targeted miRNAs of the screened prognostic lncRNAs and to identify the intersection with the differentially expressed miRNAs. Seven common miRNAs were obtained (Figure 2A). Similarly, the Starbase tool was used to analyze the targeted mRNAs of seven common miRNAs and identify the intersection with the differentially expressed mRNAs. Twenty-seven common mRNAs were obtained (Figure 2B). The screened lncRNAs, miRNAs, and mRNAs were used to construct a ceRNA co-expression network, which consisted of 40 nodes and 80 edges (Figure 2C; Table 3).

**TABLE 3:** Key lncRNAs, miRNAs, and mRNAs in the ceRNA network of lung adenocarcinoma.

lncRNAs	Binding miRNAs	Associated mRNAs
SMIM25	hsa-miR-182-5p, hsa-miR-1-3p, hsa-miR-143-3p	*TICRR*, *SLC7A11*, *NQ O 1*, *PRC1*, *CYBRD1*, *SOX5*, *TIMP3*
PCAT19	hsa-miR-378a-3p, hsa-miR-143-3p	*KIF4A*, *SAPCD2*, *ADM2*, *SLC2A1*, *HMMR*, *TICRR*, *NQ O 1*, *SCN8A*, *SLC7A5*, *E2F2*, *KRT80*, *PCDHA12*, *CAVIN1*, *GATA6*, *TBX3*, *CACNG4*, *FGF2*, *ACVRL1*, *SLC39A8*, *ABCA3*, *ARHGEF26*, *MME*
LINC00261	hsa-miR-577, hsa-miR-1269a, hsa-miR-708-5p, hsa-miR-182-5p, hsa-miR-1-3p	*SLC7A11*

### PPI Network Construction and Functional Enrichment Analysis of lncRNA-Related mRNAs in Lung Adenocarcinoma

The co-expression analysis was carried out to construct PPI networks to disclose the potential roles and mechanisms of hub lncRNAs in LUAD. Twenty-seven DEmRNAs in the ceRNA network were considered to be research objects. The co-expression analysis of lncRNA-mRNAs was performed using Starbase and GEPIA. LncRNA-mRNAs pairs with *p* < 0.05 were considered reliable (Supplementary Table S3). Then, the STRING database was used to construct a PPI network with lncRNA-associated mRNAs. In total, 38 edges and 13 nodes were involved in the PPI network (Figure 2D). One significant module containing 7 edges and 15 nodes was selected from the PPI network using the MCODE plug-in in Cytoscape (Figure 2E).

**FIGURE 3:**
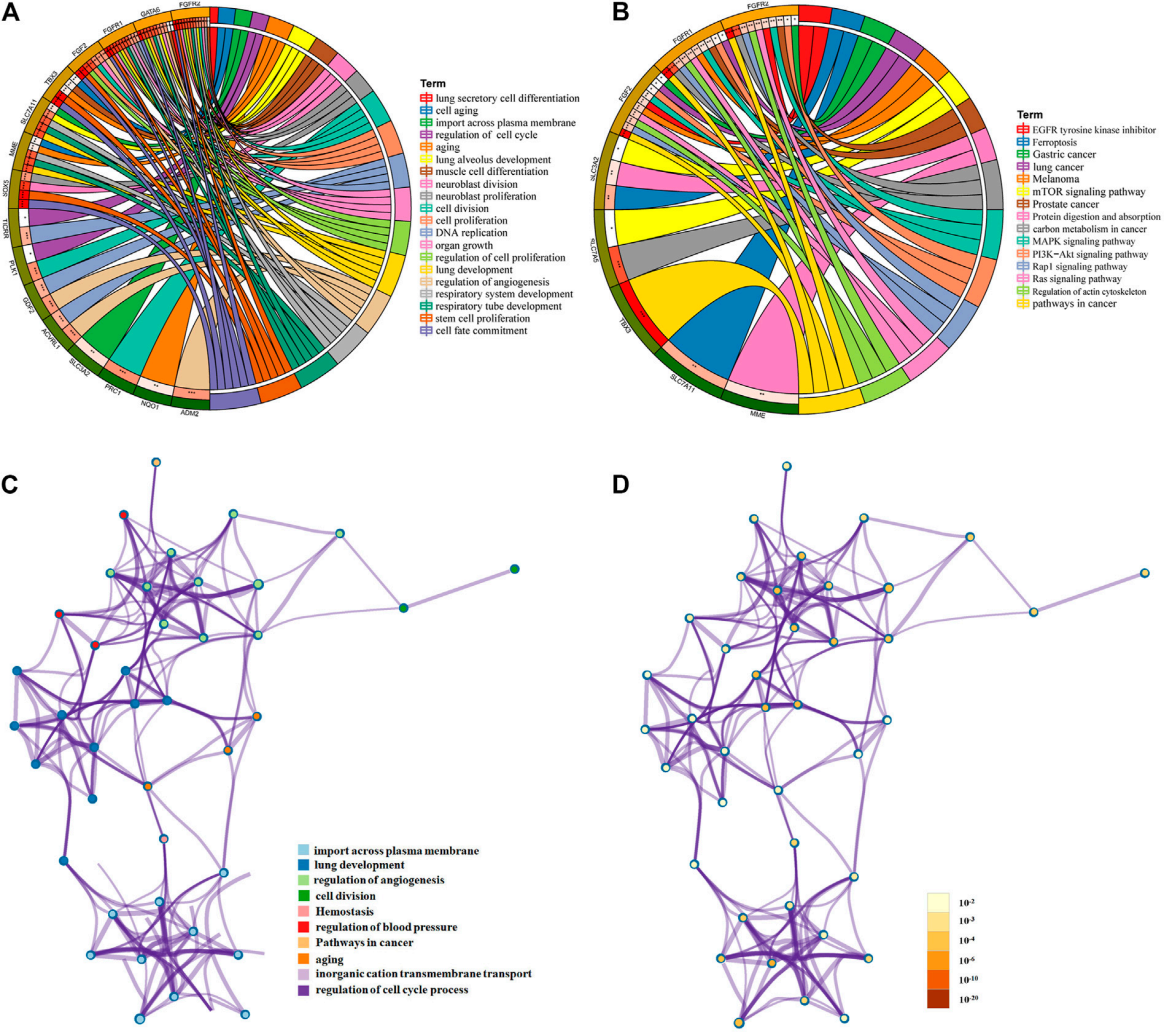
Functional and pathway enrichment analysis of mRNAs in ceRNA. Chord diagram showing enriched **(A)** GO clusters and **(B)** KEGG clusters for the mRNAs; mRNAs are shown on the left, and enriched GO or KEGG clusters are shown on the right; **(C,D)** interaction network of GO- and KEGG-enriched terms using Metascape. **(C)** Colored by cluster ID, where nodes that share the same cluster ID are typically close to each other; **(D)** colored by *p*-value, where terms containing more genes tend to have a more significant *p*-value.

GO and KEGG analyses of mRNAs in the PPI network were performed using Metascape. The results showed that these lncRNA-associated mRNAs were obviously associated with cell senescence, cell division, cell cycle, angiogenesis, transmembrane signal transduction, and lung development (Figure 3A). For KEGG pathways, pathways in cancer were the most significantly enriched (Figure 3B). All identified processes and pathways of lncRNA-associated mRNAs were connected. To further explore the relationships, a network plot of enriched terms was selected and shown (Figures 3C,D). The enriched items were distributed in concentration, and the interaction between the enriched items was conspicuous.

### Cox Analysis of lncRNAs and Validation of Hub lncRNA Expression in Clinical Samples of Lung Adenocarcinoma and Lung Cancer Cells (A549 and SPC-A1 Cells)

**FIGURE 4:**
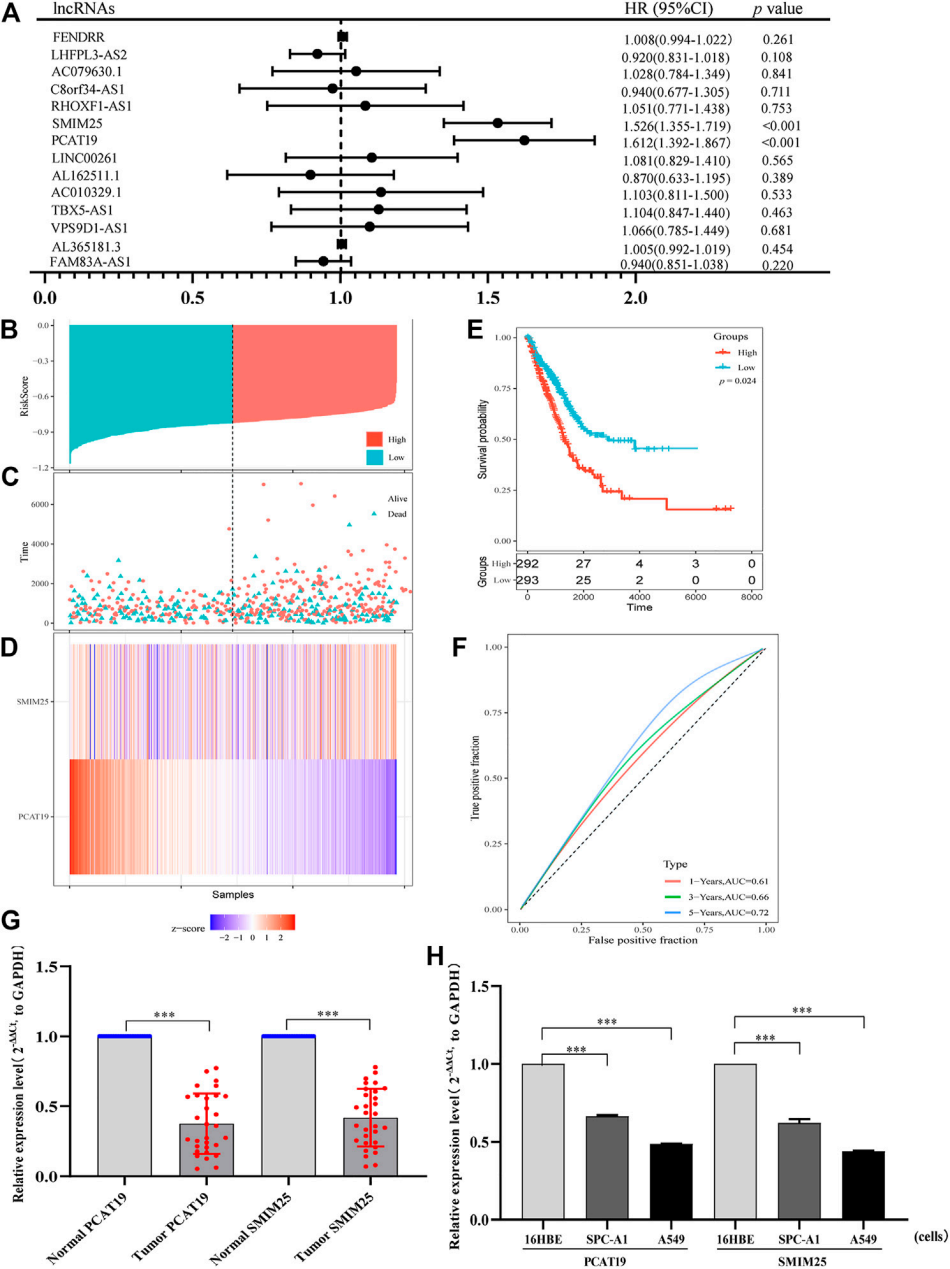
**(A)** Univariate Cox proportional hazard regression analysis of 14 lncRNAs related to the prognosis of patients with lung adenocarcinoma. **(B)** Risk score distribution, **(C)** patient survival status distribution, and **(D)** heatmap of PCAT19 and SMIM25 expression by the risk score. **(E)** Kaplan–Meier curves for high-risk and low-risk groups. **(F)** ROC curves for predicting the survival in lung adenocarcinoma patients by the risk score. **(G,H)** RT-qPCR analysis was conducted to assess the expression levels of PCAT19 and SMIM25 in **(G)** lung adenocarcinoma tissues (the control was advanced normal tissues) and **(H)** A549 and SPC-A1 cells (the control was 16HBE cells). The expression level of GAPDH was used as an internal reference, and the data are shown as the mean ± standard deviation. ***p < 0.001.

To further identify the lncRNAs related to the prognosis of LUAD patients, we conducted the univariable Cox regression analysis. As a result, we found that two lncRNAs (SMIM25 and PCAT19) were more significantly related to the prognosis of patients with LUAD (Figure 4A). Subsequently, the multivariate Cox analysis showed that SMIM25 and PCAT19 may be independent prognostic factors for LUAD (Table 4B). Then, we constructed a risk scoring model based on the expression levels of SMIM25 and PCAT19 in LUAD expression profiles from TCGA. The risk score calculation formula is as follows: risk score = (0.0156) × Exp (SMIM25) + (−0.068) × Exp (PCAT19). Based on the risk score, patients with LUAD were divided into high- and low-risk groups. The risk curve, scatter plot, and Kaplan–Meier analysis showed that the overall survival (OS) of the high-risk group was poor (Figures 4B–E). The ROC curve analysis showed that the AUCs at 1, 3, and 5 years were 0.61, 0.66, and 0.72, respectively (Figure 4F). All results show that the risk scoring model based on the expression of SMIM25 and PCAT19 has a predictive value. Therefore, the above results confirm that SMIM25 and PCAT19 might be useful candidates for LUAD patient outcome prediction.

**TABLE 4:** **(A)** Correlation between overall survival and multivariable characteristics *via* multivariate survival analysis of PCAT19 and **(B)** multivariate survival analysis of SMIM25 and PCAT19.

Clinical characteristics	HR	Lower	Upper	*P*
A				
PCAT19	0.920	0.831	0.918	0.011
Age	1.008	0.994	1.022	0.261
M	0.940	0.677	1.305	0.711
N	1.266	1.048	1.529	0.014
T	1.236	1.035	1.477	0.019
Gender	1.028	0.784	1.349	0.841
Stage	1.328	1.115	1.583	0.002
**LncRNAs**	**HR**	**Lower**	**Upper**	***P***
B				
SMIM25	1.032	0.937	1.382	0.027
PCAT19	1.124	0.729	1.029	0.011

Furthermore, we validated two prognostic lncRNAs (SMIM25 and PCAT19) in LUAD clinical tissues (normal adjacent tissues as the control) and cell line (16HBE as the control). Thirty LUAD and 30 adjacent normal tissues were detected by using qRT-PCR. As depicted in Figure 4G, SMIM25 and PCAT19 transcript levels were significantly lower in LUAD than normal tissues (*p* < 0.05). The same results were shown in A549 and SPC-A1 cells (*p* < 0.05) (Figure 4H). The expression difference of PCAT19 in the two lncRNAs was more significant; therefore, PCAT19 was selected as the follow-up research object.

### Correlation Analysis of PCAT19 Expression Level and Clinicopathological Parameters

**FIGURE 5:**
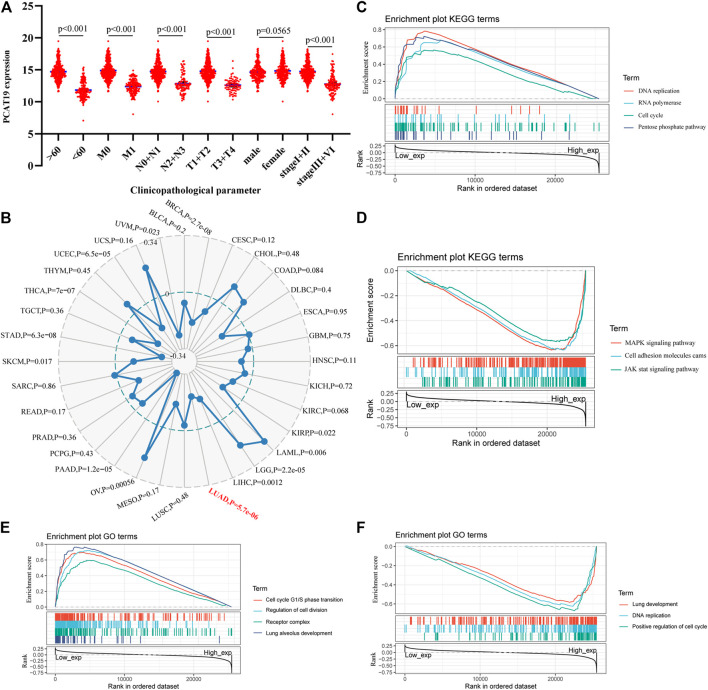
**(A)** Correlation analysis of the expression of PCAT19 with clinicopathological parameters of lung adenocarcinoma. **(B)** Radar chart shows the correlation between the expression of PCAT19 and the tumor mutational burden of each tumor. The red font represents lung adenocarcinoma; **(C,D)** KEGG pathway analysis showed **(C)** positively correlated groups and **(D)** negatively correlated groups; **(E,F)** GO term analysis revealed **(E)** positively correlated groups and **(F)** negatively correlated groups.

We evaluated the relationship between the expression level of PCAT19 and various clinicopathological parameters in patients with LUAD using data from TCGA. The low expression of PCAT19 was significantly correlated with the tumor histological grade (MNT), age and tumor stage, showing a significant negative correlation (Figure 5A, *p* < 0.05). Then, the multivariate logistic analysis using the above three parameters demonstrated that the expression level of PCAT19 (HR = 0.920, 95% CI: 0.831–1.018, *p* = 0.011), tumor histological grade (NT) (HR = 1.266, 95% CI: 1.048–1.529, *p* = 0.014, and HR = 1.236, 95% CI: 1.035–1.477, *p* = 0.019), and tumor stage (HR = 1.328, 95% CI: 1.115–1.583, *p* = 0.002) were extremely significantly associated with the overall survival of LUAD patients (Table 4A). In addition, we analyzed the relationship among PCAT19 expression, immune neoantigens, tumor mutational burden, and microsatellite instability in LUAD. The results showed that PCAT19 expression and tumor mutation burden were also significantly negatively correlated, indicating that the lower the PCAT19 expression in LUAD tumor cells, the higher the number of mutations contained (Figure 5B, *p* < 0.05). These results suggested that LUAD patients with low expression of PCAT19 were more likely to develop more advanced tumors than those with high expression of PCAT19.

In addition, we conducted a gene set enrichment analysis (GSEA) on PCAT19 to further explore the biological functions in which PCAT19 may participate. As shown in Figures 5C–F, the biological processes and molecular functions closely related to PCAT19 were mainly pneumotocele and lung development, cell circulation, and DNA repair. The KEGG pathway analysis showed that four pathways had the strongest positive correlation with PCAT19 expression: DNA repair, cell cycle, pentose phosphate pathway, and RNA polymerase. The three most negatively correlated pathways were the MAPK signaling pathway, JAK signaling pathway, and cell adsorption process.

### Effects of PCAT19 on the Proliferation, Migration, and Invasion of Lung Cancer Cells (A549 and SPC-A1 Cells)

**FIGURE 6:**
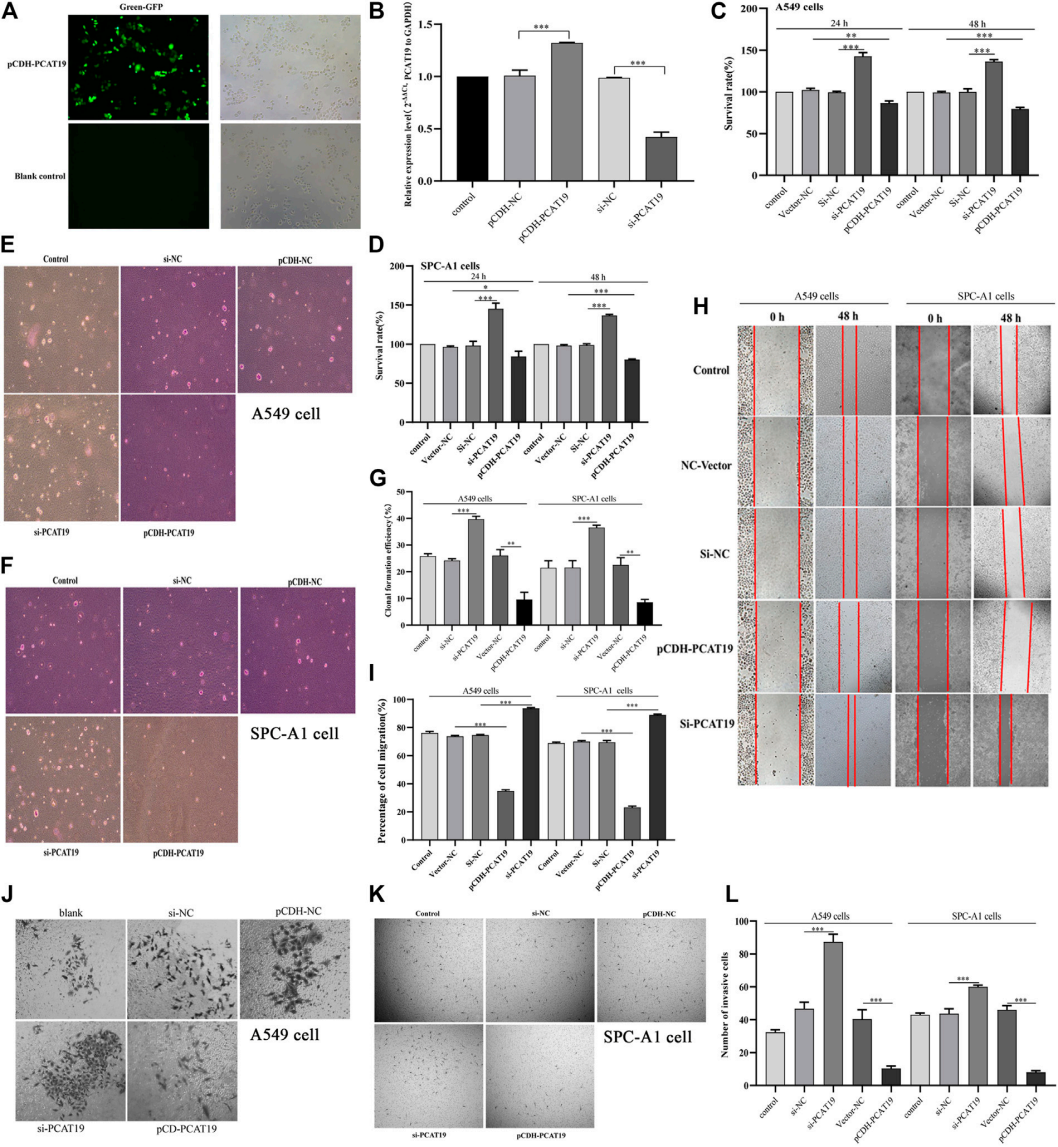
**(A)** Overexpression of PCAT19 in A549 cells analyzed by fluorescence observation; **(B)** transfection efficiency of pCDH-PCAT19 and si-PCAT19 was detected by qPCR; **(C)** effects of PCAT19 knockdown or overexpression on the vitality of A549 cells measured using the CCK-8 assay; **(D)** effects of PCAT19 knockdown or overexpression on the vitality of SPC-A1 cells measured using the CCK-8 assay; **(E–G)** effect of PCAT19 knockdown or overexpression on A549 and SPC-A1 cell proliferation measured using the soft agar assay. **(H,I)** effects of PCAT19 knockdown or overexpression on A549 and SPC-A1 cell migration measured using the migration assay. **(J–L)** effects of PCAT19 knockdown or overexpression on A549 and SPC-A1 cells invasion using transwell assays. *p < 0.05, **p < 0.01, ***p < 0.001.

To further study the role and effect of PCAT19 in A549 and SPC-A1 cells, we transfected cells with PCAT19 overexpression plasmid and siRNA to perform functional gain experiments. Fluorescence microscopy analysis of PCAT19 confirmed successful transfection of the overexpression plasmid (Figure 6A). Subsequently, the expression level of PCAT19 in cells was detected by qPCR. PCAT19 expression was significantly increased in cells transfected with PCAT19 overexpression plasmid. The cells transfected with si-PCAT19 showed an opposite trend (Figure 6B). Then, we conducted CCK-8, soft agar, wound healing, and transwell assays. CCK-8 detection results showed that PCAT19 knockdown significantly promoted the proliferation of A549 and SPC-A1 cells, and overexpression of PCAT19 inhibited the proliferation of A549 and SPC-A1 cells (Figures 6C,D). The soft agar colony formation assay showed the same trend. Overexpression of PCAT19 inhibited the formation of clones, and the number of clones in the si-PCAT19 group increased significantly (Figures 6E–G). In addition, wound healing and transwell assays showed that si-PCAT19 significantly promoted A549 and SPC-A1 cell migration and invasion compared with the control group (*p* < 0.05) (Figures 6H–L). In addition, the PCAT19 overexpression group had a poor migration distance and invasion rate than the control group. Similarly, the PCAT19 knockout group showed a greater distance and invasion rate than the control group (Figures 6I,L). These results further showed that downregulation of PCAT19 significantly promoted the migration and invasion of A549 and SPC-A1 cells, and overexpression of PCAT19 decreased the migration and invasion of A549 and SPC-A1 cells. Collectively, these results suggested that PCAT19 regulates the proliferation, migration, and invasion of A549 and SPC-A1 cells.

### MiR-143-3p Reverses the Effect of PCAT19 on the Proliferation of A549 and SPC-A1 Cells

**FIGURE 7:**
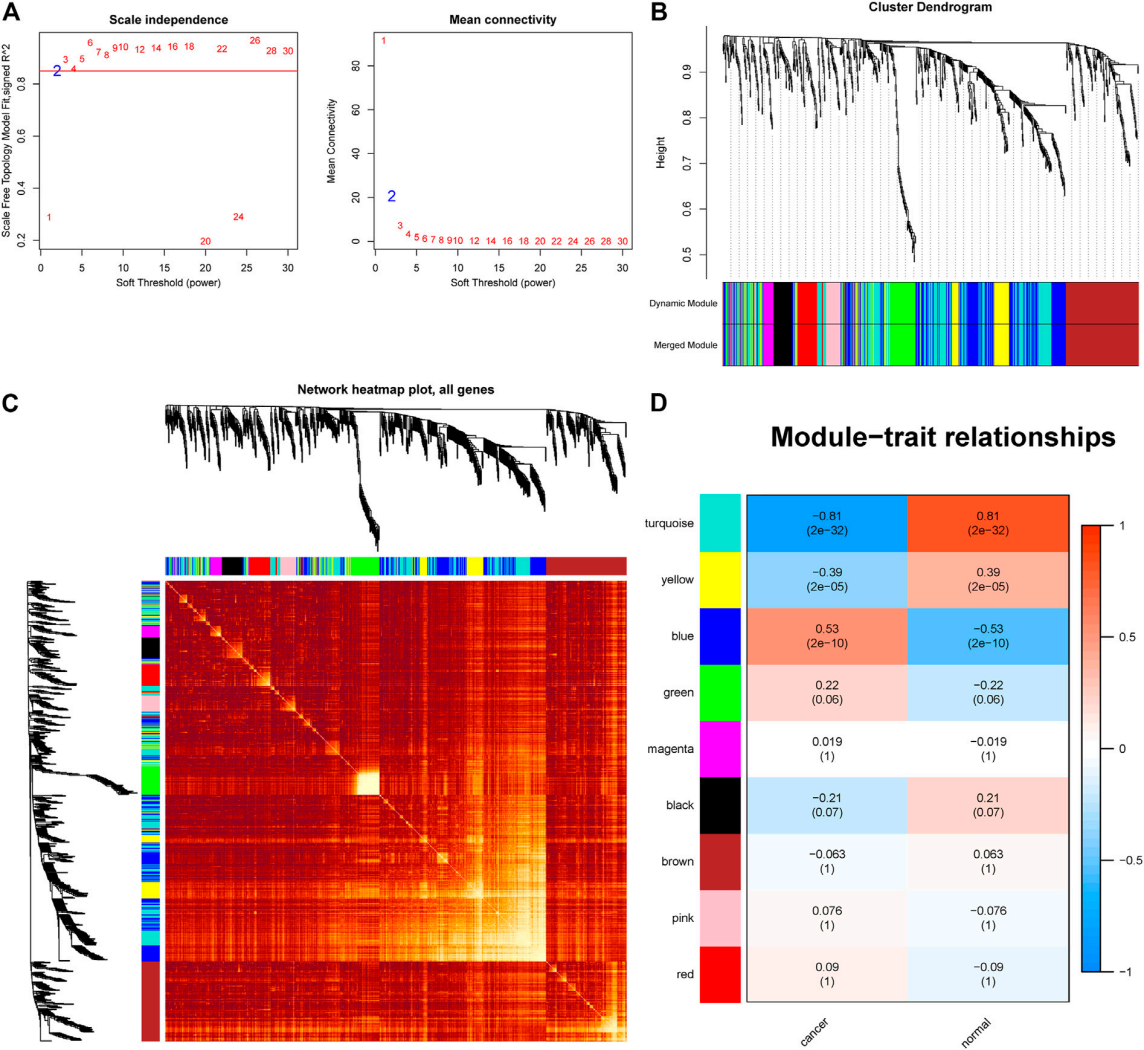
WGCNA identification of lung adenocarcinoma associated miRNA modules in GSE74190. **(A)** Soft-threshold power versus scale-free topology model fit index and mean connectivity. **(B)** Cluster dendrogram of the co-expression network modules. **(C)** Cluster dendrogram of the co-expression network modules. **(D)** Analysis of the miRNA relationship between lung adenocarcinoma and normal sample modules.

**FIGURE 8:**
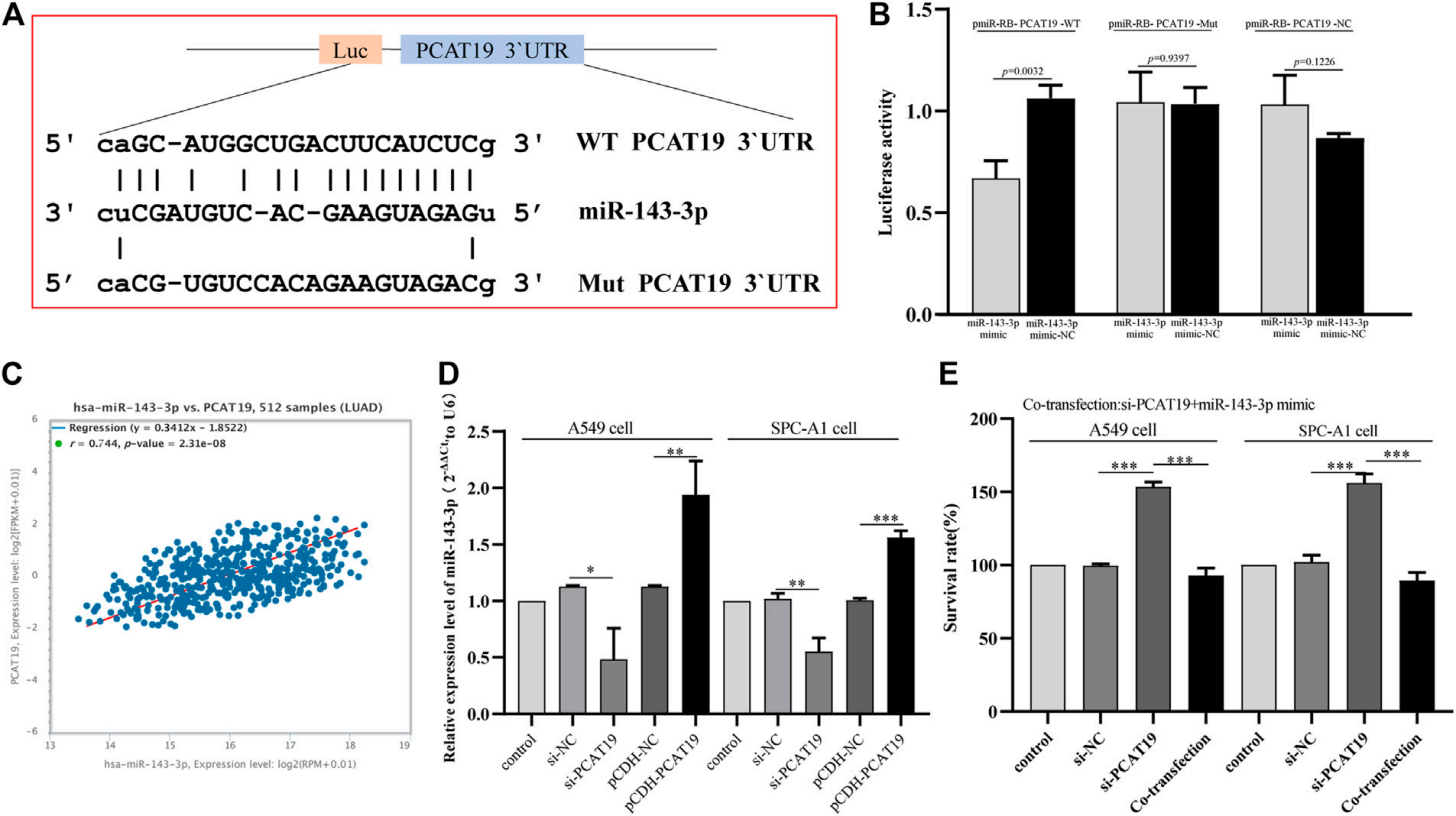
**(A)** Schematic diagram of the binding site between miR-143-3p and WT or Mut PCAT19. **(B)** Luciferase activity of WT or Mut PCAT19 after co-transfection of miR-143-3p mimic and WT or Mut PCAT19 dual fluorescent vector into A549 cells. **(C)** Correlation between the expression levels of PCAT19 and miR-143-3p. **(D)** Expression change of miR-143-3p after PCAT19 knockdown or overexpression in A549 and SPC-A1 cells. The expression level of U6 was used as internal reference. **(E)** CCK-8 analysis of the proliferation activity of A549 and SPC-A1 cells co-transfected with si-PCAT19 and miR-143-3p mimic. The data shown are the mean ± standard deviation of three experiments. *p < 0.05, **p < 0.01, ***p < 0.001.

To further study the miRNAs targeted by PCAT19, we used WGCNA to construct a co-expression network to identify hub miRNAs related to LUAD. First, we downloaded the LUAD miRNA expression profile dataset GSE74190 from the GEO database and performed WGCNA based on these data. When soft-power β was set to 2, the scale-free topology was suitable for indices exceeding 0.85 (Figure 7A). β = 2 was used to construct a hierarchical clustering tree with different colors representing different modules in LUAD samples, and nine gene modules were obtained (Figures 7B,C). As shown in Figure 7D, the turquoise module was considered to be the hub module with a correlation coefficient of 0.81 (*p* = 2e-17). Twelve hub miRNAs were further screened from the turquoise module and then intersected with the miRNAs targeted by PCAT19 in the ceRNA network. Finally, miR-143-3p was obtained (Supplementary Table S4). The dual luciferase experiment further verified that PCAT19 and miR-143-3p are directly targeted (Figures 8A,B). Correlation analysis results showed that PCAT19 and miR-143-3p were positively correlated (Figure 8C). The qPCR results showed that the expression level of miR-143-3p was increased in A549 and SPC-A1 cells overexpressing of PCAT19. Conversely, the expression level of miR-143-3p in A549 and SPC-A1 cells with PCAT19 knockout was also decreased (Figure 8D). In addition, co-transfected with si-PCAT19 and miR-143-3p mimic in A549 and SPC-A1 cells, the CCK-8 results showed that the proliferation activity of A549 and SPC-A1 cells was inhibited compared with that of the PCAT19 knockout group (Figure 8E), indicating that the miR-143-3p mimic might reverse the proliferative effect of si-PCAT19 on A549 and SPC-A1 cells.

## Discussion

LUAD is one of the leading causes of cancer-related deaths worldwide. Abnormal expression of lncRNAs can affect biological functions of cells such as tumor cell proliferation, migration, invasion, and apoptosis (Murillo-Maldonado and Riesgo-Escovar, 2019; Ginn et al., 2020). Recent studies have shown that lncRNAs play an important role in the progression of LUAD. For example, lncRNA DGCR5 promotes the proliferation of LUAD cells (Dong et al., 2018), and lncRNA TTN-AS1 promotes the migration and invasion of LUAD cells (Jia et al., 2019). lncRNA HAGLR inhibits tumor growth of LUAD by silencing E2F1 (Guo et al., 2019). lncRNA ACTA2-AS1 inhibits the progression of LUAD by increasing the expression of SOX7 (Ying et al., 2020). Existing studies have shown that the role of lncRNAs in lung cancer (including LUAD) is largely unclear, which will help understand the molecular mechanisms involved in the development of lung cancer and find therapeutic strategies. However, the reported expression pattern and molecular function are only 1%, and there are fewer characterizations of lncRNAs (Yang et al., 2014). Therefore, in-depth research on the function of unknown lncRNAs is still needed.

At present, public databases of tumor RNA expression profiles, such as TCGA and GEO, show a continuous growth trend, which indicates that these databases will become key tools for verifying and researching clinical problems. In this study, we downloaded LUAD RNA expression profile data from the TCGA database. A total of 49 differentially expressed lncRNAs were screened, and 112 differentially expressed miRNAs and 2,953 differentially expressed mRNAs were obtained. Through Kaplan–Meier curve analysis, 16 lncRNAs related to the prognosis of LUAD patients were obtained. Then, based on the differentially expressed lncRNA-miRNA co-expression relationship, the differentially expressed miRNA-mRNA co-expression relationship and targeted RNAs (lncRNAs, miRNAs, and miRNAs), a ceRNA coaction network was constructed, including 3 lncRNAs (SMIM25, PCAT19, and LINC00261), 7 miRNAs, and 27 mRNAs. Through the comprehensive analysis of lncRNA-related genes, we can better grasp the molecular functions of abnormal lncRNA expression (Ma et al., 2012; Signal et al., 2016). Therefore, we analyzed 27 mRNAs related to three lncRNAs (SMIM25, PCAT19, and LINC00261) in the ceRNA network. GO and KEGG analyses of mRNAs were mainly enriched in cell proliferation, cell cycle, transmembrane signal transduction, and lung development. The pathway in cancer was the most significantly enriched KEGG. These data indicate that three lncRNAs (SMIM25, PCAT19, and LINC00261) were involved in the important tumor-related progression of LUAD.

In addition, to further study the impact of the three selected lncRNAs on the progression of LUAD, we used univariate Cox, multivariate Cox, risk prediction models, and qPCR. The results showed that the expression of PCAT19 was more significant in LUAD tissues and was more correlated with the prognosis of LUAD patients. Low expression of PCAT19 was an independent prognostic factor of poor survival in patients with LUAD. Further analysis of PCAT19 found that the expression of PCAT19 was significantly correlated with the histological grade (TMN), age, and tumor grade of LUAD, showing a significant negative correlation. In addition, the expression of PCAT19 was also negatively correlated with the tumor mutational burden of LUAD. Studies have reported that the high expression of PCAT19 in patients with laryngeal cancer is associated with poor overall survival of patients with laryngeal cancer (Xu et al., 2019). The high expression of PCAT19 in NSCLC significantly reduces the survival rate of patients (Zhang et al., 2019). All these results indicate that PCAT19 has a certain influence on tumor progression. To further understand the influence of PCAT19 on the progression of LUAD, we first performed GSEA and found that PCAT19 was mainly involved in the process of cell function. Subsequently, functional loss-gain experiments confirmed that the low expression of PCAT19 can promote the proliferation, migration, and invasion of A549 and SPC-A1 cells. In contrast, the high expression of PCAT19 had the opposite effect. These results indicate that PCAT19 has an important regulatory effect on the progression of LUAD. Previous studies also confirmed that PCAT19 could regulate the progression of other cancers. For example, lncRNA PCAT19 promotes the proliferation of laryngocarcinoma cells *via* modulation of the miR-182/PDK4 axis (Xu et al., 2019). PCAT19 interacts with HNRNPAB to activate a subset of cell-cycle genes associated with prostate cancer progression, thereby promoting prostate cancer tumor growth and metastasis (Hua et al., 2018). Additionally, PCAT19 negatively regulates the p53 tumor-suppression pathway, promoting cancer cell proliferation in patients with NSCLC (Zhang et al., 2019).

Moreover, to further understand the regulatory process of PCAT19, we obtained the coacting pairs (PCAT19/miR-143-3p) of PCAT19 through miRNAs in WGCNA and ceRNA. A dual luciferase assay confirmed the direct targeting relationship between PCAT19 and miR-143-3p, and the expression levels of PCAT19 and miR-143-3p were positively correlated, suggesting that PCAT19 may play a sponge role of miR-143-3p in LUAD. miR-143-3p has been proven to play an important role in many tumors. For example, miR-143-3p inhibits the tumorigenesis of pancreatic ductal adenocarcinoma by targeting KRAS (Xie et al., 2019); MiR-143-3p targets MAPK7 to inhibit the proliferation, migration, and invasion of osteosarcoma cells (Hou et al., 2019); LINC00667/miR-143-3p regulates RRM2 to affect the progression of small-cell lung cancer cells (Yang et al., 2019). The results of co-transfection experiments show that overexpression of miR-143-3p can reverse the effect of PCAT19 knockout on the proliferation of A549 and SPC-A1 cells, which further illustrates the regulatory relationship between PCAT19 and miR-143-3p, indicating that PCAT19/miR-143-3p plays an important role in the progression of LUAD.

However, limitations still existed in this study. For example, because we examined only 30 patients, our results may be accidental and need to be confirmed in large LUAD clinical cases. In addition, the mechanism of the PCAT19 regulation axis has not been further studied in our research. Even so, the trend should be analogous. Taken together, our study proves that PCAT19 may stimulate the development of LUAD and possesses great potential to be a prognostic biomarker and therapeutic target for LUAD.

## Conclusion

In summary, using TCGA RNA sequencing data of LUAD, combined with ceRNA, PPI, GO and KEGG analyses, Cox regression analysis, risk model, and qPCR, the independent prognostic factor lncRNA PCAT19 for LUAD patients was screened. Cell function experiments confirmed that PCAT19 can affect proliferation, migration, and invasion of LUAD cells. In addition, the direct targeting RNA miR-143-3p of PCAT19 was screened in WGCNA and dual luciferase experiments. Overexpression of miR-143-3p reversed the effect of PCAT19 knockout on the proliferation of LUAD cells. In short, PCAT19 can be used as a potential biomarker for predicting LUAD.

## Data Availability

The original contributions presented in the study are included in the article/Supplementary Material, and further inquiries can be directed to the corresponding authors.
