# Supplementary material for: Integrated Dissection of lncRNA-miRNA-mRNA Pairs and Potential Regulatory Role of lncRNA PCAT19 in Lung Adenocarcinoma
Source: Front Genet. 2022 Jan 12;12:765275. doi: 10.3389/fgene.2021.765275 (PMC8790230; doi:10.3389/fgene.2021.765275)
Supplement: Supplementary file 2 [file Table8.DOCX]

supplementary table 4. Hub miRNAs in WGCNA core module and miRNs in ceRNA network.

|  | **miRNAs** |
| --- | --- |
| Hub miRNAs in turquoise module | miR-154, miR-323-3p, miR-376b, miR-379, miR-381, miR-382, miR-409-3p, miR-410, **miR-143-3p**, miR-493, miR-495, **miR-577**. |
| miRNs in ceRNA network | **miR-143-3p**, miR-708-5p, miR-182-5p, **miR-577**, miR-1-3p, miR-1269a, miR-378a-3p. |

Black and bold fonts indicate the same miRNAs.
