# Supplementary figures and images for: Integrated Dissection of lncRNA-miRNA-mRNA Pairs and Potential Regulatory Role of lncRNA PCAT19 in Lung Adenocarcinoma
Source: Front Genet. 2022 Jan 12;12:765275. doi: 10.3389/fgene.2021.765275 (PMC8790230; doi:10.3389/fgene.2021.765275)

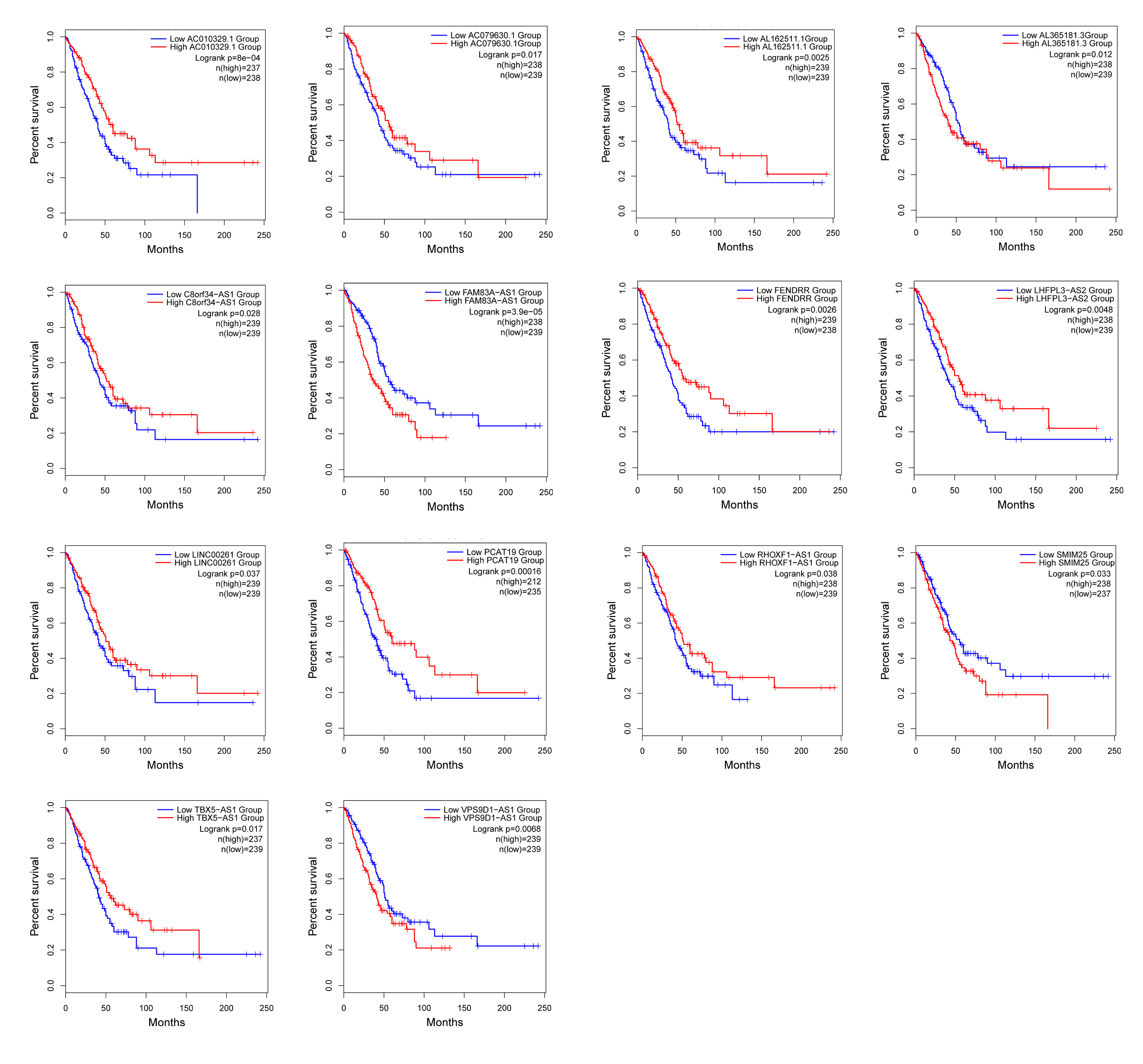

Supplement: Supplementary file 4 [file Image1.JPEG]

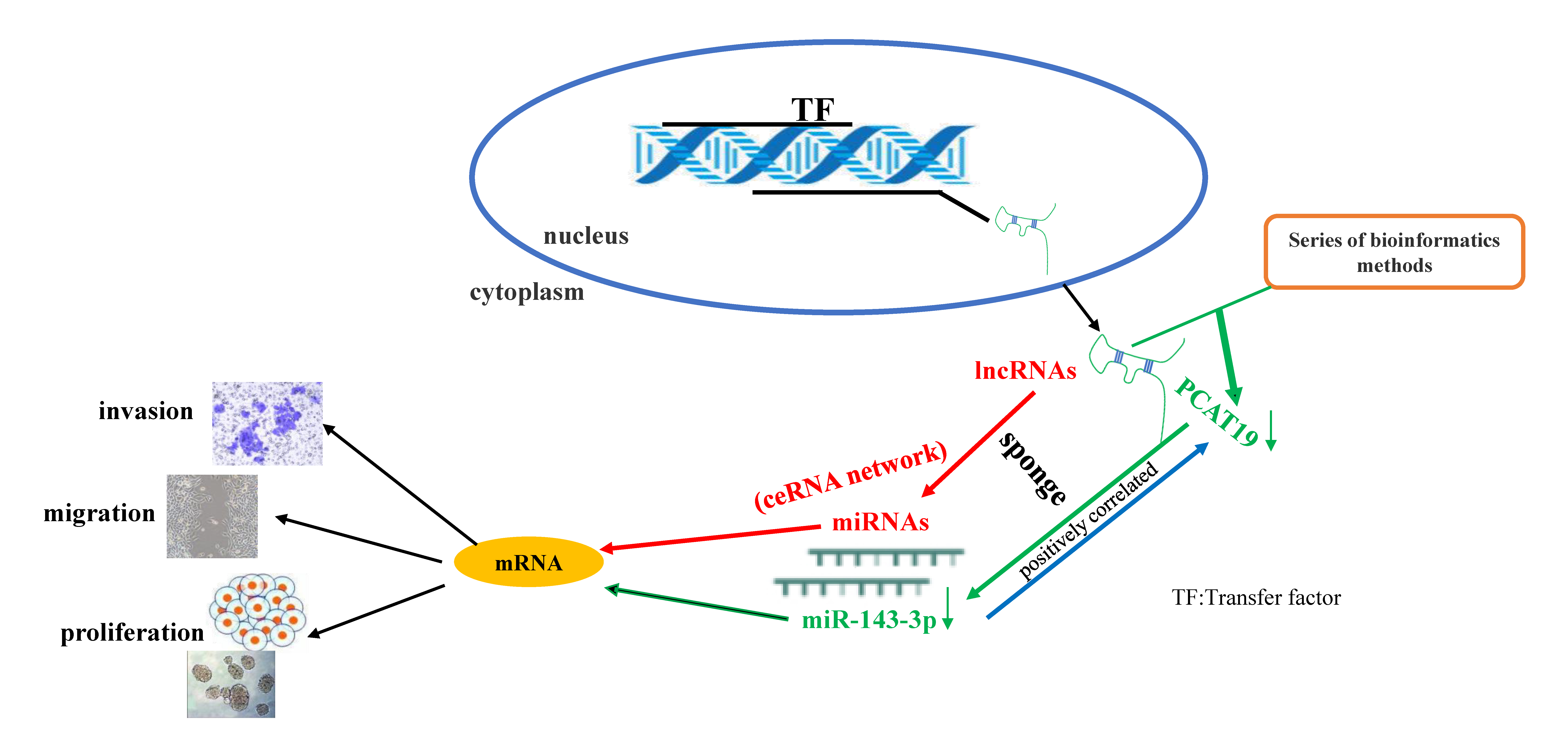

Supplement: Supplementary file 6 [file Image2.JPEG]
