# Supplementary material for: Integrated Dissection of lncRNA-miRNA-mRNA Pairs and Potential Regulatory Role of lncRNA PCAT19 in Lung Adenocarcinoma
Source: Front Genet. 2022 Jan 12;12:765275. doi: 10.3389/fgene.2021.765275 (PMC8790230; doi:10.3389/fgene.2021.765275)
Supplement: Supplementary file 5 [file Table7.DOCX]

Supplementary table 3. Co-expression analysis of associated mRNAs and lncRNAs in ceRNA.

| **associated mRNAs** | **lncRNAs-mRNA co-expression** | | | | | |
| --- | --- | --- | --- | --- | --- | --- |
|  | **SMIM25** | | **PCAT19** | | **LINC00261** | |
|  | *r* | *p*-value | *r* | *p*-value | *r* | *p*-value |
| *KIF4A* | -6.50E-02 | 1.34E-01 | -2.50E-01 | 6.18E-09 | -4.97E-01 | 3.11E-34 |
| *SAPCD2^*^* | -1.22E-01 | 5.14E-03 | -2.32E-01 | 7.01E-08 | -3.85E-01 | 5.50E-20 |
| *ADM2* | 4.40E-02 | 3.13E-01 | -4.50E-02 | 2.98E-01 | -3.00E-03 | 9.42E-01 |
| *SLC2A1* | -1.00E-03 | 9.75E-01 | -2.28E-01 | 1.19E-07 | -4.15E-01 | 2.52E-23 |
| *HMMR* | -2.20E-02 | 6.19E-01 | -2.07E-01 | 1.65E-06 | -4.29E-01 | 5.52E-25 |
| *TICRR^*^* | -1.53E-01 | 4.33E-04 | -2.36E-01 | 4.31E-08 | -4.78E-01 | 2.07E-31 |
| *NQO1* | -1.27E-01 | 3.49E-03 | -7.90E-02 | 7.07E-02 | 9.20E-02 | 3.51E-02 |
| *SCN8A* | -2.20E-01 | 3.27E-07 | -7.60E-02 | 8.20E-02 | 1.70E-02 | 6.89E-01 |
| *SLC7A5^*^* | -9.50E-02 | 2.88E-02 | -1.49E-01 | 6.25E-04 | -3.49E-01 | 1.62E-16 |
| *E2F2* | -2.00E-02 | 6.55E-01 | -1.78E-01 | 4.04E-05 | -3.70E-01 | 1.69E-18 |
| *KRT80* | 5.80E-02 | 1.83E-01 | -1.03E-01 | 1.79E-02 | -3.36E-01 | 2.63E-15 |
| *PCDHA12* | 2.60E-02 | 5.53E-01 | 4.10E-02 | 5.52E-01 | 9,80E-02 | 2.49E-02 |
| *CAVIN1* | 3.78E-01 | 2.80E-19 | 3.14E-01 | 1.57E-13 | 5.10E-02 | 2.44E-01 |
| *GATA6* | 7.70E-02 | 7.84E-02 | 2.77E-01 | 1.02E-10 | 3.57E-01 | 3.03E-17 |
| *TBX3^*^* | 1.30E-01 | 2.72E-03 | 4.04E-01 | 4.11E-22 | 2.17E-01 | 4.88E-07 |
| *CACNG4* | 8.00E-02 | 6.76E-02 | 9.00E-02 | 3.80E-02 | -6.00E-03 | 8.99E-01 |
| *FGF2^*^* | 1.13E-01 | 9.64E-03 | 3.63E-01 | 7.88E-18 | 1.49E-01 | 5.84E-04 |
| *ACVRL1^*^* | 5.34E-01 | 4.67E-40 | 5.89E-01 | 1.68E-50 | 1.63E-01 | 1.74E-04 |
| *SLC39A8^*^* | 3.13E-01 | 2.10E-13 | 1.95E-01 | 6.61E-06 | 2.38E-01 | 3.22E-08 |
| *ABCA3^*^* | 1.81E-01 | 3.07E-05 | 2.31E-01 | 8.20E-08 | 4.05E-01 | 3.68E-22 |
| *ARHGEF26^*^* | 1.49E-01 | 6.02E-04 | 1.82E-01 | 2.70E-05 | 1.19E-01 | 6.42E-03 |
| *MME^*^* | 2.64E-01 | 7.49E-10 | 1.24E-01 | 4.32E-03 | -1.02E-01 | 1.94E-02 |
| *PRC1^*^* | -1.30E-01 | 2.88E-03 | -2.86E-01 | 2.20E-11 | -4.73E-01 | 9.68E-31 |
| *CYBRD1^*^* | 2.19E-01 | 4.09E-09 | 3.47E-01 | 2.39E-16 | 3.15E-01 | 1.43E-13 |
| *SOX5 ^*^* | 9.00E-02 | 3.90E-02 | 3.39E-01 | 1.20E-15 | 2.57E-01 | 2.13E-09 |
| *TIMP3* | 3.14E-01 | 1.69E-13 | 1.45E-01 | 8.87E-04 | -1.60E-02 | 7.16E-01 |
| *SLC7A11* | -2.38E-01 | 3.19E-08 | -4.80E-02 | 2.74E-01 | -1.00E-02 | 8.25E-01 |

“*” represents key lncRNA-associated.
