# Supplementary material for: Integrated Dissection of lncRNA-miRNA-mRNA Pairs and Potential Regulatory Role of lncRNA PCAT19 in Lung Adenocarcinoma
Source: Front Genet. 2022 Jan 12;12:765275. doi: 10.3389/fgene.2021.765275 (PMC8790230; doi:10.3389/fgene.2021.765275)
Supplement: Supplementary file 8 [file Table5.DOCX]

Supplementary table 1. Top10 (up- and down-regulated) of differentially expressed miRNAs in normal tissues and lung adenocarcinoma tissues.

| **symbol** | **logFC** | **AveExpr** | **t** | **PValue** | **FDR** |
| --- | --- | --- | --- | --- | --- |
| **Up regulation** |  |  |  |  |  |
| hsa-miR-31-5p | 3.0389 | 1.3220 | 7.1555 | 2.59E-12 | 9.65E-12 |
| hsa-miR-301a-5p | 3.1004 | 1.4914 | 11.9006 | 2.63E-29 | 3.68E-28 |
| hsa-miR-577 | 3.1182 | 1.1828 | 8.8484 | 1.13E-17 | 6.30E-17 |
| hsa-miR-1287-3p | 3.1721 | 2.4670 | 14.8126 | 3.36E-42 | 1.67E-40 |
| hsa-miR-3607-3p | 3.3181 | 4.4861 | 10.3209 | 5.31E-23 | 4.17E-22 |
| hsa-miR-196a-5p | 3.5011 | 3.4533 | 5.9436 | 4.87E-09 | 1.41E-08 |
| hsa-miR-153-5p | 3.5306 | 2.9001 | 12.1356 | 2.73E-30 | 4.69E-29 |
| hsa-miR-1269a | 3.8065 | 2.2325 | 5.5674 | 3.99E-08 | 1.08E-07 |
| hsa-miR-9-5p | 3.9566 | 9.2710 | 10.4150 | 2.32E-23 | 1.89E-22 |
| hsa-miR-210-3p | 5.0760 | 9.0656 | 18.1944 | 1.34E-58 | 1.49E-56 |
| **Down regulation** |  |  |  |  |  |
| hsa-miR-184 | -4.1874 | 1.2219 | -15.2241 | 3.96E-44 | 2.21E-42 |
| hsa-miR-486-5p | -4.1287 | 6.7484 | -18.6387 | 7.89E-61 | 1.18E-58 |
| hsa-miR-139-3p | -3.4530 | 3.3611 | -19.3149 | 3.02E-64 | 6.75E-62 |
| hsa-miR-30c-2-3p | -3.3257 | 4.7679 | -19.9349 | 2.12E-67 | 9.46E-65 |
| hsa-miR-144-5p | -3.0231 | 6.3113 | -12.3336 | 3.97E-31 | 7.39E-30 |
| hsa-miR-1247-3p | -2.9490 | 2.9335 | -11.6477 | 2.93E-28 | 3.97E-27 |
| hsa-miR-30a-3p | -2.9200 | 11.8096 | -16.2908 | 3.18E-49 | 2.37E-47 |
| hsa-miR-144-3p | -2.8254 | 2.7860 | -12.0912 | 4.20E-30 | 6.95E-29 |
| hsa-miR-451a | -2.7600 | 8.6431 | -11.5573 | 6.88E-28 | 8.78E-27 |
| hsa-miR-133a-3p | -2.5516 | 2.2218 | -11.9869 | 1.15E-29 | 1.71E-28 |
