# Supplementary material for: Integrated Dissection of lncRNA-miRNA-mRNA Pairs and Potential Regulatory Role of lncRNA PCAT19 in Lung Adenocarcinoma
Source: Front Genet. 2022 Jan 12;12:765275. doi: 10.3389/fgene.2021.765275 (PMC8790230; doi:10.3389/fgene.2021.765275)
Supplement: Supplementary file 10 [file Table6.DOCX]

Supplementary table 2. Top10 (up- and down-regulated) of differentially expressed mRNAs in normal tissues and lung adenocarcinoma tissues.

| **symbol** | **logFC** | **AveExpr** | **t** | **p-Value** | **FDR** |
| --- | --- | --- | --- | --- | --- |
| **Up regulation** |  |  |  |  |  |
| ABCA12 | 4.6920 | 1.9938 | 11.1424 | 2.78E-26 | 3.21E-25 |
| SYT12 | 4.7383 | 2.6760 | 9.2391 | 4.55E-19 | 3.16E-18 |
| MMP11 | 4.8481 | 4.6751 | 9.5715 | 2.92E-20 | 2.22E-19 |
| COL11A1 | 5.1211 | 3.5159 | 7.6978 | 5.92E-14 | 2.77E-13 |
| MMP13 | 5.1850 | 1.8198 | 9.6710 | 1.27E-20 | 9.92E-20 |
| PRAME | 5.1963 | 0.4641 | 8.3867 | 3.74E-16 | 2.11E-15 |
| CYP24A1 | 5.1998 | 3.5932 | 7.2312 | 1.50E-12 | 6.23E-12 |
| PITX2 | 5.5066 | -0.2655 | 11.6046 | 3.62E-28 | 4.88E-27 |
| CST1 | 6.0597 | 1.0043 | 11.2864 | 7.27E-27 | 8.77E-26 |
| FAM83A | 6.1222 | 5.3632 | 10.5563 | 5.77E-24 | 5.66E-23 |
| **Down regulation** |  |  |  |  |  |
| SFTPC | -8.5100 | 6.9391 | -10.3642 | 3.19E-23 | 2.97E-22 |
| CLDN18 | -6.9744 | 3.7801 | -17.7016 | 1.78E-56 | 9.36E-55 |
| LGI3 | -6.8238 | 0.2749 | -31.8073 | 1.09E-129 | 4.24E-126 |
| AGER | -6.6183 | 5.0967 | -20.3513 | 4.98E-70 | 4.68E-68 |
| FABP4 | -6.1851 | 1.3985 | -36.2175 | 1.38E-151 | 2.14E-147 |
| GPM6A | -5.9488 | 0.4727 | -35.5822 | 1.72E-148 | 1.33E-144 |
| SCGB1A1 | -5.8189 | 3.6632 | -12.4005 | 1.61E-31 | 2.65E-30 |
| UPK3B | -5.7805 | 1.9851 | -30.5464 | 2.89E-123 | 3.21E-120 |
| ANKRD1 | -5.6522 | 0.4838 | -29.6023 | 2.05E-118 | 1.77E-115 |
| GKN2 | -5.5214 | 0.6278 | -20.8589 | 1.14E-72 | 1.22E-70 |
